# DATIV—Remote Enhancement of Smart Aerosol Measurement System Using Raspberry Pi-Based Distributed Sensors

**DOI:** 10.3390/s24134314

**Published:** 2024-07-02

**Authors:** Gazi Hasanuzzaman, Tom Buchwald, Christoph Schunk, Christoph Egbers, Andreas Schröder, Uwe Hampel

**Affiliations:** 1 Department of Aerodynamics and Fluid Mechanics, Brandenburg University of Technology Cottbus-Senftenberg (BTU C-S), 03046 Cottbus, Germany; 2 Chair of Image Based Measurement Techniques, Brandenburg University of Technology Cottbus-Senftenberg (BTU C-S), 03046 Cottbus, Germany; 3 Institute of Fluid Dynamics, Helmholtz-Zentrum Dresden-Rossendorf (HZDR), 01328 Dresden, Germany; 4 Department of Experimental Methods, Institute of Aerodynamics and Flow Technology, German Aerospace Center (DLR), 37073 Göttingen, Germany; 5 Chair of Imaging Techniques in Energy and Process Engineering, Dresden University of Technology (TUD), 01069 Dresden, Germany

**Keywords:** particulate matter, aerosol, COVID-19, distributed sensors, Raspberry Pi, WiFi, open source, low-cost measurement system, indoor ventilation

## Abstract

Enclosed public spaces are hotspots for airborne disease transmission. To measure and maintain indoor air quality in terms of airborne transmission, an open source, low cost and distributed array of particulate matter sensors was developed and named Dynamic Aerosol Transport for Indoor Ventilation, or DATIV, system. This system can use multiple particulate matter sensors (PMSs) simultaneously and can be remotely controlled using a Raspberry Pi-based operating system. The data acquisition system can be easily operated using the GUI within any common browser installed on a remote device such as a PC or smartphone with a corresponding IP address. The software architecture and validation measurements are presented together with possible future developments.

## 1. Introduction

The emergence of COVID-19 demonstrated the catastrophic impact of airborne respiratory diseases. COVID-19 and similar airborne diseases can cause long-term health problems and significant disruption to social and economic life. The most common modes of transmission for similar diseases are droplet/particulate matter (PM) transmission, direct contact and airborne transmission via small aerosol particles [1]. However, during the COVID-19 pandemic, pathogens in respiratory aerosol particles were the dominant mode of transmission. According to the World Health Organization [2], respiratory particles larger than 5 μm are considered droplets and smaller particles are considered aerosols. Studies [3] have found similar aerosol size distributions in cough aerosols and exhaled breath from patients with respiratory infections, with most pathogens concentrated in small particles (<5 μm). There is considerable disagreement among experts regarding the average particle size of respiratory aerosols. However, numerous studies have demonstrated that they represent the primary mode of virus transmission indoors [4,5,6]. This is because small aerosol particles have the potential to be transmitted to a multitude of individuals at a considerable distance from the source of infection. This is due to their lack of settling by gravity, which allows them to remain suspended in the air and accumulate over time. This renders them more susceptible to aerodynamic transport. Consequently, indoor environments such as classrooms, meeting rooms, clubs, etc., are susceptible to the spread of viruses due to the presence of long-lived, small aerosols (with a diameter of less than 5 µm) when inadequate or disproportionate ventilation is apparent.

Small aerosol particles can carry many viruses depending on the location of the infection in the respiratory tract of the spreader [7]. The same study suggested that COVID-19 RNA are also present in respiratory aerosols with mean diameters of 0.25∼0.5 μm. Furthermore, they have a greater potential to cause a bronchial infection. Once in the susceptible host, they tend to be inhaled deeply, leading to potential infection in the lower airways, while larger droplets tend to be retained in the upper airways. Consequently, the mitigation strategies employed in indoor environments should be directed toward reducing the number of small aerosol particles.

The effects of preventive measures, such as different types of masks, air purifiers and air ventilation, within group dynamic scenarios are scarcely investigated and need proper attention [8]. Particularly, smaller particles pose a greater threat due to their ability to infiltrate beyond normal masks. An experimental study by [9] on the effectiveness of different masks concluded that simple surgical or homemade masks do not provide sufficient protection against small droplets with diameters of 0.3–2 μm. Nevertheless, the use of such masks is recommended when no particle-filtering respiratory mask is available.

When making recommendations for indoor infection control measures, it is usually assumed that the “well mixed” hypothesis [10] applies. This hypothesis postulates that particles are rapidly dispersed throughout the indoor air due to thorough turbulent mixing, resulting in a homogeneous distribution. Despite adherence to infection control measures based on the well-mixed hypothesis, infections can still occur when ventilation transfers airborne particles from one infected person to another, as confirmed by real-life case studies by [11,12]. In order to ascertain the specific circumstances under which the well-mixed hypothesis does not hold, it is necessary to conduct large-scale measurements. Lagrangian particle tracking approaches, such as Shake-The-Box, are well-suited to this task as they are capable of accurately measuring flow fields in large measurement volumes.

In [13], measurements using two-dimensional Shake-The-Box Lagrangian particle tracking are presented, revealing the flow topologies in a cross-section of a 4.2 m × 2.8 m classroom. They studied transient scenarios with dynamic motion of the occupants. Such experiments require expensive equipment, such as large LED arrays and high-resolution cameras, but provide insight into the Lagrangian and Eulerian properties of such large flow domains. Nevertheless, this approach does not permit the assessment of infection risk at each location in arbitrary room geometries and the influence of human motion on aerosol transport due to limitations in optical accessibility.

In order to implement an effective indoor ventilation strategy, it is necessary to study different transient scenarios. This includes the simulation of several realistic boundary conditions, such as windows, enclosed space areas, forced/natural ventilation mechanisms, continuous and/or intermittent ventilation ducts, indoor/outdoor temperature difference, likely host/carrier location, etc. The large degree of freedom therefore requires a high number of experiments, which is only possible with a simple experimental approach. The measurement of aerosol particle concentration fulfills this requirement when a flexible, remote and robust measurement system is available for the simultaneous measurement of aerosol particle concentration at multiple locations.

The monitoring of indoor air quality for pollutants using PMS is usually carried out according to reference standards. The same principle is applied to respiratory aerosols. This is associated with high capital cost and large size, making it unsuitable for smart array or PMS devices. In order to design an efficient, intelligent and low-cost measurement system, a number of requirements must be taken into account, such as compact size for easy installation, low power consumption for longer operation, accessibility, low-cost production and simultaneous array operation. However, it is important to note that small and inexpensive optical particle counters are now readily available and easy to use. Nevertheless, it has been shown that such devices give different mass concentration measurements than reference devices and are sensitive to humidity [14].

In [15], a performance study was conducted to evaluate different consumer-grade particulate matter sensors under controlled air pollution and thermal conditions. Four PM sensors, Sensirion SPS30 (Stäfa, Switzerland), Alphasense OPC-R1, Alphasense OPC-N3 (Essex, United Kingdom) and Nova Fitness SDS018 (Jinan, China), were used in the test. These sensors, coupled with an Arduino Mega, transmitted and logged PM data to a personal computer. Five indoor air pollutant sources producing different particle diameter spectra ranging from ≤0.1 μm (ultrafine particles) to <10 μm (coarse particles) with concentrations from 2 ppm to 2387 ppm were used to simulate PM levels. The results showed that all sensors underestimated the mass concentration of particulate matter. However, the SPS30 showed a strong correlation with the results obtained by the reference measurement system, a Grimm model 1371, Durag Aerosol Technik miniWRAS (Hamburg, Germany). In contrast to the other consumer sensors tested, the Pearson correlation coefficient was greater than 0.8 for each particle source. The results show that the SPS30 is an effective sensor for detecting changes in PM concentration in the range of 0.3–2.5 μm.

In recent years, a number of distributed, low-cost PM measurement systems have been developed and deployed. One example is a measurement system development reported in the study by [16] which is capable of integrating a variety of sensors, including the Sensirion SPS30 PMS. Such measurement systems are suitable for experiments in a variety of room geometries and boundary conditions. Recently, experiments based on PMSs have been utilized to measure the particle dispersion in an aircraft cabin [17]. The SPS30 used in this study can also deviate significantly from reference instruments, such as the Grimm 1.108, but with very low inter-instrument variability [18]. Therefore, they are still suitable for aerosol particle transport experiments as long as only one type is used. However, these systems rely on interconnecting cables between the sensors and a host computer and are therefore limited for experiments with widely separated measurement points or moving occupants.

In order to record the dispersion of particles in specific situations and spatial geometries as part of experiments, sensors are required at several locations in the room to record the particle concentration, which can then be read out. Such a system has been developed by [19] and based on a low-cost PM sensor (HPMA115S0) which has been used to monitor indoor air quality in controlled experiments with incense, burnt smoke and toast particles. Additional instruments, such as the TSI Model 3938 Scanning Mobility Particle Sizer (SMPS) and Model 3321 Aerodynamic Particle Sizer (APS), were used to calculate measurements with coefficients of determination (R2) ranging from 0.21 to 0.99 for PM2.5. An indoor environmental monitoring device based on Arduino Pro Mini was developed to measure relative humidity, temperature, CO_2_ concentration, motion and PM concentration [20]. This device, known as the School Monitoring Box (SKOMOBO), included various sensors and interfaces for external devices.

Weekly et al. (2013) [21] presented a wireless PM sensor for the detection of coarse airborne particulate matter in indoor environments. The device used PPD-20V and DSM301A sensors to detect PM with different size ranges. Data from the PM sensor were remotely transmitted to a computer for analysis, with calibration using a GT-526S laser particle counter. The results of the experiment demonstrated the effectiveness of the device in detecting coarse particles and thus correlating the PM data with the occupant concentration in the public spaces. The idea of using PM concentration in relation to the number of people is a novel approach, but the system is purely static and deals with the number of people rather than PM concentration data.

However, both sensor systems described are based on custom-designed boards and the software required to operate them is not freely available. Therefore, we present an open source, low-cost and distributed PM measurement system designed to measure dynamic scenarios such as DATIV. Thus, we present a distributed measurement system equipped with essential features to perform aerosol dispersion experiments in realistic settings with moving participants. The system’s affordability and simplicity make it suitable not only for extensive use in experiments investigating the transmission of virus-laden aerosols, but also for general assessment of air quality in different indoor environments.

## 2. Methodology

### 2.1. Hardware

Using low-cost hardware such as the Raspberry Pi Zero 2W (Cambridge, United Kingdom) and consumer-grade networking hardware for portable measurement systems offers cost-effectiveness and easy scalability, making it an ideal choice for a wide range of data collection applications. Raspberry Pi’s extended General Purpose Input/Output (GPIO) ports make it easy to integrate sensors and peripherals. Moreover, they have many options to add other sensors besides the SPS30. They come with an I2C bus, SPI bus, UART, CSI and they also have full USB connectivity. This makes it easier to install and integrate various other sensors. These single board computers (SBCs) are readily available, inexpensive, customizable and benefit from a robust community. The widespread adoption of the Raspberry Pi has spurred the emergence of a competitive market for alternative SBC platforms. When designing our measurement platform, we prioritized portability to ensure that our software could be seamlessly adapted to a variety of alternative hardware platforms. The Raspberry Pi devices can run Python scripts or other programming languages to process the sensor data. Therefore, in addition to supporting the Raspberry Pi, we prioritized portability to other SBC platforms to avoid potential supply chain issues that became a crisis point during the COVID-19 pandemic.

**Figure 1:**
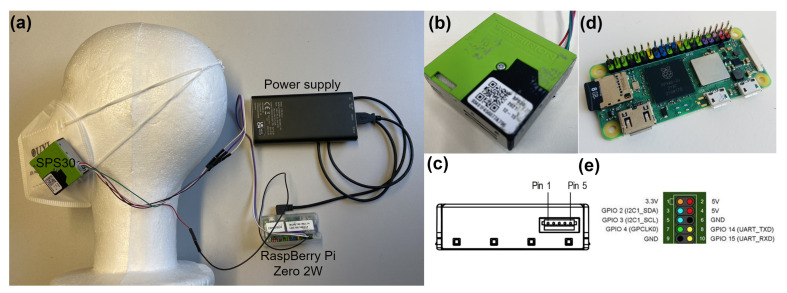
(**a**) Portable DATIV system where an SBC is powered by a DC power supply; (**b**) The SPS30 acts as the PMS and transmits via the computer; (**c**) The communication interface connector is located on the side of the sensor opposite to the air outlet; (**d**) The Raspberry Pi Zero acts as the computer, hosting the measurement software and communicating via an internal antenna within the WiFi domain to transmit the data; (**e**) The color-coded built-in GPIO of the computer.

While Figure 1a shows the simple and portable setup of the DATIV system, Figure 1b–e [22] show the SPS30 PMS, the Raspberry Pi Zero 2W single board computer and the built-in internal color interpretation of the GPIO on the Raspberry Pi Zero.

The presented measurement system is capable of acquiring particle images and of computing a particle number concentration. However, as previous experiments have shown [8], the use of small particle counters for this purpose is preferable. Therefore, the focus of this contribution is the measurement of the local concentration of aerosol particles with Sensirion’s SPS30 [23]. These sensors are compact and small in size (41×41×12 mm^3^), making them the perfect choice for developing remote measurement systems such as DATIV. The PMS is based on the principle of an optical particle counter (OPC) using laser scattering. Air is drawn into the cell through a small inlet by a small fan. The incoming air is then passed through a laser beam which is scattered by the particles in the air. The scattered light is detected by a photo-diode, which converts the number of scattered particles into a digital signal, providing the SBC with real-time particle count and mass concentration values. While OPCs perform similarly when counting particles, the algorithm that converts the signals to particle concentration differentiates between the sensor manufacturers. In addition to the sensor algorithm, the optical parameters of the particles, such as refractive index and shape, have a significant effect on particle estimation. As a result, laser-based measurements are inherently different from more accurate reference systems that use direct weight-based methods.

The number concentration of particles is given as #/cm^3^. For indoor air with PM1 where ultrafine particles (<1 μm) are present, this sensor can measure particles between 0.3 μm and 1 μm with 10% concentration accuracy. If the number concentration is higher than 3000 #/cm^3^, the accuracy is not specified. In addition, the sensor operates at a uniform sampling rate of 1±0.04 s. The accuracy of each SPS30 PM sensor depends on the calibration accuracy, which refers to the output deviation of an individual sensor compared to the average output of a group of sensors during calibration. Typically, this accuracy is affected by factors such as aerosol homogeneity, environmental conditions, sensor stability and the stability of the calibration reference. Therefore, accuracy is defined as the deviation of the output of an individual sensor from the average output of a batch of sensors within specified operating conditions such as 25 °C and nominal supply voltage, e.g., 5 V. For the present experiment, where PM1 aerosol particles were used, the sensor has a mass concentration accuracy of ±10 μg/m^3^ and maximum long-term mass concentration precision limit drift of ±1.25 μg/m^3^ per year with an operating condition of 0 to 100 μg/m^3^ aerosol concentration. Overall, the DATIV system has the accuracy of the installed SPS30 sensors.

On the opposite side of the SPS30 air outlet, there are five single communication connectors, as shown in Figure 1c from pin 1∼5. Of these, 1, 2, 3 and 4 must be connected to the SBC GPIO, as shown in red in Figure 1e. The communication ports of the SPS30 (corresponding to the SBC GPIO number) correspond to 1. DC power connector for 5 V ±10% (4), 2. data receive pin (8), 3. transmit pin (10), 4. interface select (not connected to GPIO) and 5. ground/GND (6). To reduce the number of pins on the GPIO, the Universal Asynchronous Receiver/Transmitter (UART) protocol was used.

To provide the system with a portable power supply, as shown in Figure 1a, each system was equipped with an Ansmann Power Bank (Model: 1700-0114). This lithium polymer battery can deliver 3.7 V at 15 Ah, giving a capacity of 55.5 Wh [24]. This means that, depending on the capacity of the power supply, DATIV can be operated for several hours at a time.

### 2.2. Network Architecture

**Figure 2:**
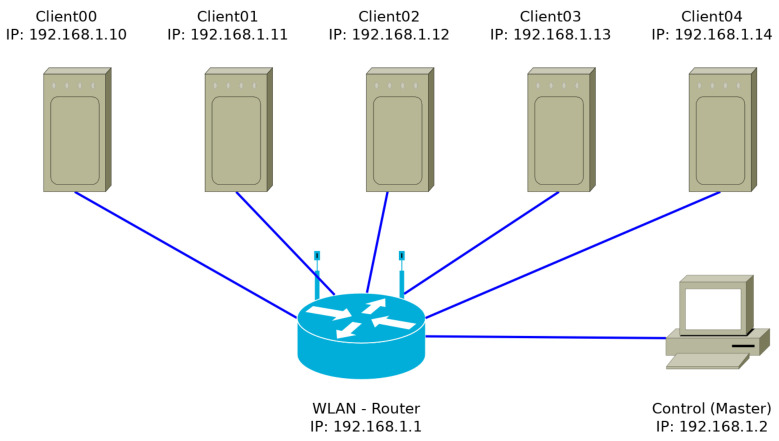
The network architecture of the sensor system consists of several SBC clients, a router and a central control computer.

The network architecture as presented in Figure 2 consists of a router and a group of portable Raspberry Pis with a power supply that allows for non-stationary measurements. Each Raspberry Pi is connected to an SPS30, which allows for communication via the UART protocol. The router acts as the central connectivity hub of the network and ensures that traffic is routed appropriately. Each measurement station has the capacity to act as a central station, enabling the transmission of messages to other units, allowing the operator to control multiple units simultaneously, initiate or terminate measurements and configure sensor parameters. In addition, each measurement unit can act as a broadcaster, initiating or terminating measurements and configuring sensor parameters. The existing version of the Raspberry Pi is capable of integrating both a camera and a particulate matter sensor. Each individual Raspberry Pi has the ability to process its collected data locally.

### 2.3. Software Architecture

**Figure 3:**
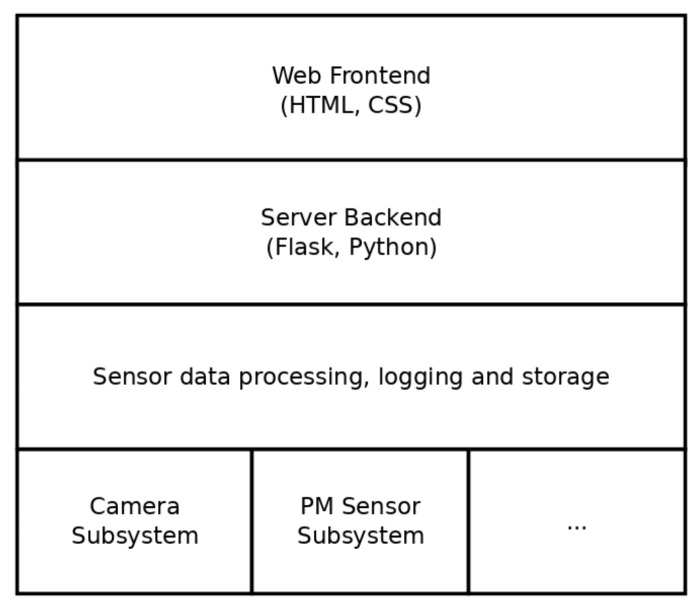
Layer diagram of the software architecture. Consists of a web front end a server back end and a framework to handle several sensor types.

To facilitate data acquisition, we have developed a dedicated software system to complement our portable measurement devices. The system is presented as a layer diagram in Figure 3. This Python-based software runs as a server process and is automatically started when the Raspberry Pi is booted. It is managed by systemd, a service management system designed for Linux-based operating systems. Systemd plays a crucial role in initializing and running services, daemons and other processes during system startup. Services are described using simple text-based service files that specify dependencies, timers, logging and automatic restart procedures in the event of a process failure. Once started, the Python-based server begins listening on an HTTP port, making it accessible via a web browser. The Python service itself uses the Flask micro web framework, which is renowned for its lightweight nature, making it an optimal choice for small-to-medium-sized applications and the development of RESTful web APIs. Flask integrates a modern template engine based on Jinja2 and an integrated development server, simplifying the development and testing phases and enabling rapid iteration. The application is divided into a front-end and a back-end component. The front end uses HTML and CSS generated from Jinja templates. These pages host custom user interfaces for controlling the sensors. In addition, the application includes API endpoints that provide the ability to control its functionality through external requests. These requests include tasks such as initializing sensors, initiating and terminating asynchronous recording and setting parameters for the sensors. Each sensor within the system provides a specific user interface depending on its particular capabilities. During the sensor data acquisition process, each sensor generates a continuous stream of readings, which are then stored in dedicated logging or data files.

**Figure 4:**
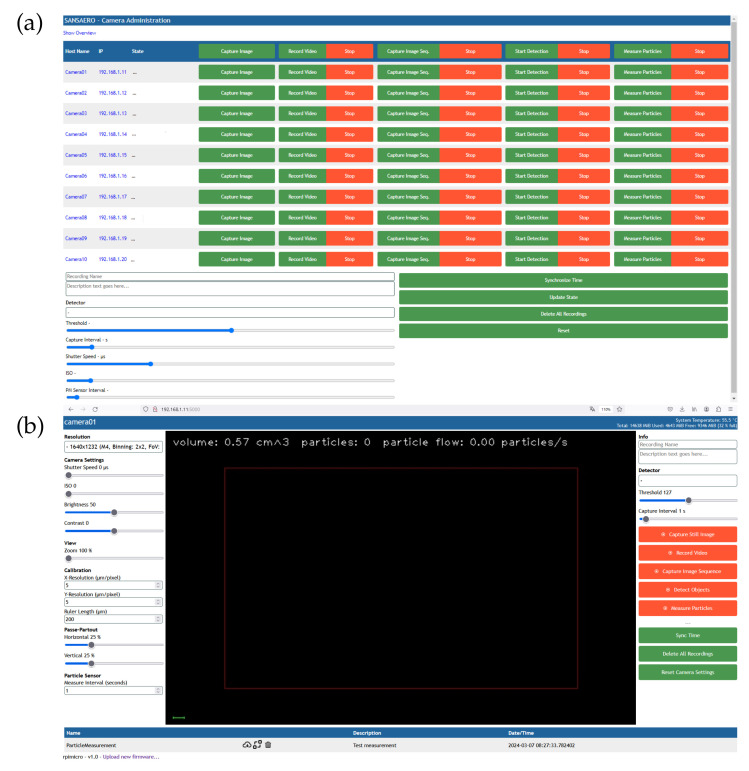
(**a**) Master GUI for all sensors, (**b**) GUI for each sensor inputs.

The Guided User Interface (GUI) can be accessed using the IP address 192.168.1.11 on port 5000 from any standard browser. Therefore, entering http://192.168.1.11 will display the master GUI with multiple sensor inputs. However, in order to access the master GUI using the previous link is possible once the DATIV system is online. The screenshot of the GUI is shown in Figure 4a. Within the Master GUI, the sensor inputs are displayed with their corresponding IP address, functionality status and various other setting parameters. DATIV has been designed for compatibility with remote image-based particle counting and PMS operation. Therefore, the system can be operated with both measurement techniques. However, in the reported functional test, only the last two columns were used for PMS operation. The PMS interval can be used as a common response time setting for all input SPS30 sensors operated within the master GUI. It is also possible to selectively operate individual sensors to start the measurement. At the end of the measurement, each sensor can also be selectively disabled. ‘Camera01’, …, ‘Camera10’ indicate the number of sensors connected to the system. However, the reported functional test presented data from three SPS30 sensors operating simultaneously. Although the figure only shows ten sensor inputs, the number of sensors can be increased and adjusted. The IP address assigned to each sensor input is also shown in the next column of sensors.

After completion of the measurement, each sensor input can be accessed from the Master GUI to the individual Sensor GUI, as shown in Figure 4b, after selection. The sensor GUI contains the adjustment parameters for both image-based and SPS30 sensor-based particle concentration measurements. The center of the GUI displays the image screen for image-based particle count measurement. Furthermore, the measurement interval for each SPS30 can be selected here. Finally, measurement data can also be downloaded/deleted from the lower part of this GUI. In order to understand the GUI operation, detailed instructions can be found from the software user manual.

## 3. Functional Test

The measurement system has been developed primarily for aerosol dispersion experiments in enclosed spaces. Therefore, this section describes a validation experiment on aerosol dispersion using the developed measurement system. The validation experiment was performed at the Cottbus Aerosol Reference Experimental (CARE) facility located at the Brandenburg University of Technology, Cottbus-Senftenberg (BTU C-S) in Cottbus, Germany, where a classroom scenario with multiple occupants was simulated. The CARE experimental classroom has a rectangular shape with a volume of approximately 130 m^3^. Further detailed information on the CARE facility is given in [8].

Two hypothetical scenarios based on one person releasing infectious aerosol particles during a 45-min lesson in a semi-populated classroom, as was the case during the COVID-19 pandemic, were investigated. In the first scenario, no ventilation was employed. In the second scenario, all windows were opened for a period of five minutes following a 20-min interval. This was one of the mitigation strategies employed in schools during the COVID-19 pandemic. The model spreader uses DEHS particles to represent aerosol particles with a diameter of less than 5 μm, which can remain suspended in the air for long periods of time. DEHS is a standard material used in experimental fluid mechanics as a tracer particle for imaging measurement techniques because it closely follows the air flow. It is therefore suitable as a model aerosol particle to mimic small diameter saliva particles, which also follow the flow topology. The experiment used a Topas ATM 230 aerosol generator to deliver particles with a mean diameter from 0.1 to 0.5 μm. The exhalation volume flow rate was 8 L/min, corresponding to the respiratory minute volume of quietly seated occupants. For further details on the DEHS particle, interested readers are referred to [25].

**Figure 5:**
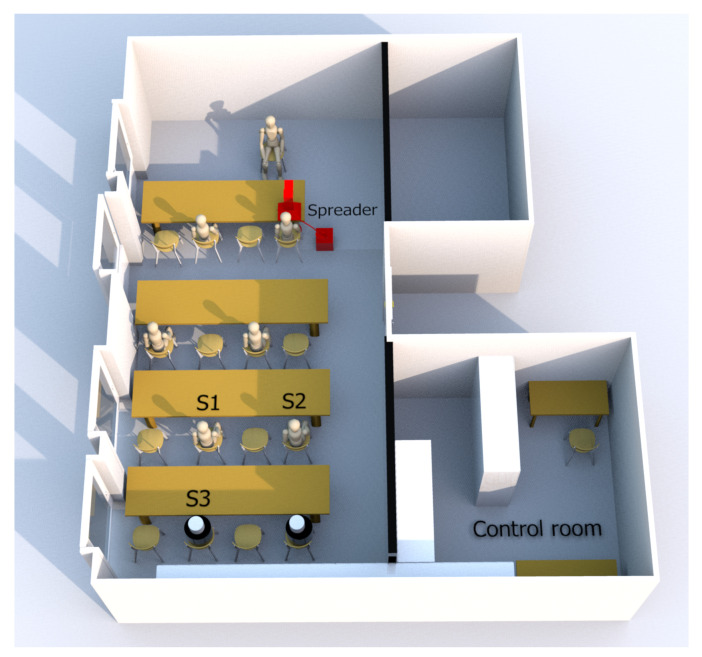
Sketch of the spreader (red) and sensor positions (S1, S2, S3) in the classroom populated by humans visualized as mannequins and heated dummies visualized as cylinders (adopted and edited from [13]).

Since the flow topology in the classroom determines the particle transport paths, it is necessary to model the heat input of the people responsible for creating a mixed convective flow. As illustrated in Figure 5, a seating arrangement comprising one teacher and eight students was employed to simulate a scenario in which a classroom was half-populated, a common occurrence during the COVID-19 pandemic. A total of seven test persons participated in the experiment, who were presented as mannequins in the figure. They were equipped with masks to which the SPS30 sensors were attached. If the test persons are not expected to move during the experiment, it is possible to replace them with dummies exhibiting a heat signature that closely resembles that of humans. Consequently, in the final row, two dummies are positioned instead of humans. These comprise a black polyethylene cylinder with a lid that serves as thermal insulation and a styropor head at which a SPS30 sensor is attached. A 75 watt light bulb was attached to each of these cylinders as an energy input. The temperature on the outside of the cylinder stabilizes at 37 °C within 40 min, which is the typical heat output for people at rest in seating position. During the spreading process, the aerosol concentration at the back of the room was measured at three different positions indicated as S1, S2 and S3, as shown in Figure 5. As the distance between the spreader in the front of the room to the sensors is high, it is expected that the well-mixed-hypothesis holds such that the aerosol particles will be relatively homogeneously distributed at the back of the room. Consequently, the measurement setup allows for the validation of the measurement accuracy by comparing the measurement results of the sensors.

### Results

**Figure 6:**
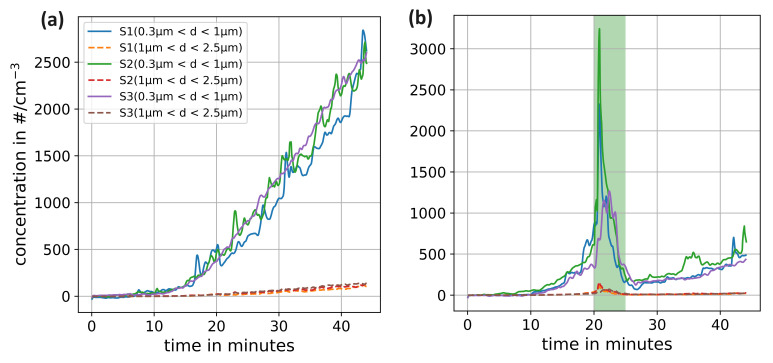
Number concentration of particles with a diameter between 0.3 μm < d < 1 μm as well as 1 μm < d < 2.5 μm of three mobile sensors S1, S2 and S3 located in the back of a classroom during an artificial spreading situation (**a**) without any ventilation and (**b**) with opening the windows for five minutes indicated by the green background.

The sensors were first synchronised using the GUI shown in Figure 4 and then instructed to start measuring simultaneously. The result downloaded from each individual sensor at the end of both experiments is shown in Figure 6a,b. As Section 3 discussed the detailed measurement conditions, Figure 6a presents the particle count where no ventilation was applied and Figure 6b shows the particle count with window opening for certain time periods.

The concentration of particles with a diameter of 0.3 μm < d < 1 μm increases throughout the measurement uniformly at all sensor positions, as expected in the classroom under investigation. A small proportion of the DEHS particles produced are larger than 1 μm, so the concentration of particles with a diameter of 1 μm < d < 2.5 μm also increases.

The results show that it takes just over 10 min for some of the exhaled particles to spread around the room and reach the back row of seats. It can be observed that extended measurements work without data loss and support the suitability of the measurement system for long-term experiments, such as the analysis of transient situations wherein the well-mixed hypothesis is not applicable. Since particle sizes can be analyzed separately, such experiments can potentially be used to study not only the dispersal of the smallest aerosol particles, but also those that should sink to the ground within a few meters, but can travel much further due to the altered flow topology caused by thermal plumes. The concentration curves depicted in Figure 6b illustrate that the opening of the window initially results in elevated particle concentrations, which then fall rapidly so that a clear reduction in concentration can be observed at the end. The increase in concentration after 20 min may be attributed to the movement of people necessary to open the windows. As previous experiments [8] suggest, this movement leads to greater mixing and thus transports more particles to the sensor positions.

When analysing the concentration, it is important to note that not all particles in the room come from the artificial source used. To obtain an accurate measurement, the particle concentration before the particle generator is switched on should be subtracted. However, transient additional sources such as pollen can present a challenge. When such external sources are identified in fresh air exchange experiments, sensors should be placed both indoors and outdoors to accurately assess their influence.

## 4. Conclusions

Large public spaces are hotspots for airborne disease transmission. To test and maintain indoor air quality for airborne transmission, an open source, low-cost and distributed particulate matter sensor (PMS) measurement system was developed and tested. This system can deploy multiple PMSs simultaneously and can be remotely controlled using the widely available Raspberry Pi-based operating system. The hardware is based on readily available low-cost components and does not require the design or manufacture of a custom board. The cost of assembling each unit is less than EUR 100, which enables the simultaneous measurements on a high number of measurement locations. The data acquisition system can be easily operated using the GUI in any common browser installed on a remote device such as PCs, tablets and/or smartphones with a corresponding IP address. The software for remote operation is simple and open source.

In the classroom scenario under investigation, in which a spreader continuously exhales particles for 45 min, it was demonstrated that the SPS30 sensors are capable of reliably measuring the increase in concentration. This corroborates the previous finding cited in reference [15], according to which changes in concentration are reliably detected by these devices. Previous experiments, as cited in [8], which demonstrated a decrease in particle concentration during spreading experiments with open windows, were successfully replicated. Furthermore, the operation and data acquisition were demonstrated to be robust over the entire 45-min period, allowing for the execution of realistic, transient experiments over the complete relevant time frame.

Consequently, the system represents the most straightforward and cost-effective approach for conducting aerosol transport studies. It is also suitable for experiments using artificial saliva, so that effects such as particle deposition, evaporation and droplet agglomeration are captured in the measurements. However, the SPS30 can only measure up to 2.5 μm and extrapolate 10 μm under appropriate conditions, so it is not suitable for measuring rapidly settling droplets.

## 5. Outlook

In particular, this software architecture can be used in applications such as environmental monitoring, home automation or industrial control. The aerosol concentration input from various remote sensors can be used as an air quality measure. The current version of the software only supports cameras and PMSs as sensor inputs. With future improvements to the software architecture, it is possible to unify the driver interface for different types of sensors, such as temperature sensors, humidity sensors, light sensors, motion detectors, air quality sensors, etc. As a result, the software is not limited to the Raspberry Pi-based platform. It is also compatible with any SBC using a Linux-based operating system and can run on the Python platform. In addition, its network-based architecture makes it easy to monitor multiple measurement units and parameters simultaneously. The latest version of the DATIV software, version 1.0 (DOI: 10.14278/rodare.2662) can be found in the master branch of the github repository at the following URL: https://rodare.hzdr.de/record/2662 (accessed on 23 April 2024) With the added benefit of portability, the application of Artificial Intelligence (AI)-based physics-informed neural networks (PINNs) similar to that presented in [26], it is also possible to integrate the DATIV system for enhanced data-based PM detection. Using the real-time monitoring capability of the DATIV system, the algorithm can continuously analyze sensor data and provide timely alerts or predictions of PM levels in the domain of distributed sensors. The system, therefore, has the potential to provide a proactive measurement capability to reduce airborne disease transmission, thereby protecting public health in indoor environments.

## Data Availability

Present version of DATIV version 1.0 software can be downloaded using the following link: https://rodare.hzdr.de/record/2662 (accessed on 23 April 2024).

## References

[1] Eykelbosh A. (2021). Indoor CO_2_ Sensors for COVID-19 Risk Mitigation: Current Guidance and Limitations.

[2] World Health Organization Modes of Transmission of Virus Causing COVID-19: Implications for IPC Precaution Recommendations. https://www.who.int/news-room/commentaries/detail/modes-of-transmission-of-virus-causing-covid-19-implications-for-ipc-precaution-recommendations.

[3] Fennelly K.P. (2020). Particle sizes of infectious aerosols: Implications for infection control. Lancet Respir. Med..

[4] Morawska L., Cao J. (2020). Airborne transmission of SARS-CoV-2: The world should face the reality. Environ. Int..

[5] Lednicky J.A., Lauzard M., Fan Z.H., Jutla A., Tilly T.B., Gangwar M., Usmani M., Shankar S.N., Mohamed K., Eiguren-Fernandez A. (2020). Viable SARS-CoV-2 in the air of a hospital room with COVID-19 patients. Int. J. Infect. Dis..

[6] Wang C.C., Prather K.A., Sznitman J., Jimenez J.L., Lakdawala S.S., Tufekci Z., Marr L.C. (2021). Airborne transmission of respiratory viruses. Science.

[7] Liu Y., Ning Z., Chen Y., Guo M., Liu Y., Kumar Gali N., Sun L., Duan Y., Cai J., Westerdahl D. (2020). Aerodynamic analysis of SARS-CoV-2 in two Wuhan hospitals. Nature.

[8] Merbold S., Hasanuzzaman G., Buchwald T., Schunk C., Schmeling D., Volkmann A., Schröder A., Egbers C. (2023). Reference experiment on aerosol particle transport for dynamic situations. Tech. Mess..

[9] Kähler J., Hain R. (2020). Fundamental protective mechanisms of face masks against droplet infections. J. Aerosol. Sci..

[10] Riley E.C., Murphy G., Riley R.L. (1978). Airborne spread of measles in a suburban elementary school. Am. J. Epidemiol..

[11] Li Y., Qian H., Hang J., Chen X., Cheng P., Ling H., Wang S., Liang P., Li J., Xiao S. (2021). Probable airborne transmission of SARS-CoV-2 in a poorly ventilated restaurant. Build. Environ..

[12] Lu J., Gu J., Li K., Xu C., Su W., Lai Z., Zhou D., Yu C., Xu B., Yang Z. (2020). COVID-19 Outbreak Associated with Air Conditioning in Restaurant, Guangzhou, China, 2020. Emerg. Infect. Dis..

[13] Buchwald T., Hasanuzzaman G., Merbold S., Schanz D., Egbers C., Schröder A. (2023). Large-scale flow field and aerosol particle transport investigations in a classroom using 2D-Shake-The-Box Lagrangian Particle Tracking. Heliyon.

[14] Stoll D., Kerner M., Paas S., Antonyuk S. (2023). Suitability of Low-Cost Sensors for Submicron Aerosol Particle Measurement. Appl. Syst. Innov..

[15] Demanega I., Mujan I., Singer B.C., Andelković A.S., Babich F., Licina D. (2021). Performance assessment of low-cost environmental monitors and single sensors under variable indoor air quality and thermal conditions. Build. Environ..

[16] Niehaus K., Westhoff A. (2022). An open-source data acquisition system for laboratory and industrial scale applications. Meas. Sci. Technol..

[17] Schmeling D., Shishkin A., Schiepel D., Wagner C. (2023). Numerical and experimental study of aerosol dispersion in the Do728 aircraft cabin. Ceas Aeronaut. J..

[18] Kuula J., Mäkelä T., Aurela M., Teinilä K., Varjonen S., González Ó., Timonen H. (2020). Laboratory evaluation of particle-size selectivity of optical low-cost particulate matter sensors. Atmos. Meas. Tech..

[19] Wang Y., Boulic M., Phipps R., Chitty C., Moses A., Weyers R., Cunningham C. (2017). Integrating open-source technologies to build a school indoor air quality monitoring box (SKOMOBO). Proceedings of the 4th Asia-Pacific World Congress on Computer Science and Engineering (APWC on CSE).

[20] Weyers R., Jang-Jaccard J., Moses A., Wang Y., Boulic M., Chitty C., Phipps R., Cunningham C. Low-Cost Indoor Air Quality (IAQ) Platform for Healthier Classrooms in New Zealand: Engineering Issues. Proceedings of the 4th Asia-Pacific World Congress on Computer Science and Engineering (APWC on CSE).

[21] Weekly K., Rim D., Zhang L., Bayen A.M., Nazaroff W.W., Spanos C.J. Low-Cost Coarse Airborne Particulate Matter Sensing for Indoor Occupancy Detection. Proceedings of the 2013 IEEE International Conference on Automation Science and Engineering (CASE).

[22] Zakaria R. (2016). Smart Motion Detection: Security System Using Raspberry Pi. J. Eng. Res..

[23] Sensirion AG Sensor Specification Statement (2020, March). https://sensirion.com/media/documents/B7AAA101/61653FB8/Sensirion_Particulate_Matter_AppNotes_Specification_Statement.pdf.

[24] Ansmann Ansmann 15Ah PD Powerbank LiPo 15000 mAh. https://asset.conrad.com/media10/add/160267/c1/-/de/002227118DS00/datablad-2227118-ansmann-15ah-pd-powerbank-lipo-15000-mah-1700-0114.pdf.

[25] Hasanuzzaman G., Merbold S., Motuz V., Egbers C. (2022). Enhanced outer peaks in turbulent boundary layer using uniform blowing at moderate Reynolds number. J. Turbul..

[26] Hasanuzzaman G., Eivazi H., Merbold S., Egbers C., Vinuesa R. (2023). Enhancement of PIV measurements via physics-informed neural networks. Meas. Sci. Technol..

